# Forkhead box O1 transcription factor; a therapeutic target for diabetic cardiomyopathy

**DOI:** 10.3389/jpps.2024.13193

**Published:** 2024-08-14

**Authors:** Tanin Shafaati, Keshav Gopal

**Affiliations:** ^1^ Faculty of Pharmacy and Pharmaceutical Sciences, University of Alberta, Edmonton, AB, Canada; ^2^ Alberta Diabetes Institute, University of Alberta, Edmonton, AB, Canada; ^3^ Cardiovascular Research Institute, University of Alberta, Edmonton, AB, Canada

**Keywords:** FoxO1, diabetic cardiomyopathy, energy metabolism, oxidative stress, cell death

## Abstract

Cardiovascular disease including diabetic cardiomyopathy (DbCM) represents the leading cause of death in people with diabetes. DbCM is defined as ventricular dysfunction in the absence of underlying vascular diseases and/or hypertension. The known molecular mediators of DbCM are multifactorial, including but not limited to insulin resistance, altered energy metabolism, lipotoxicity, endothelial dysfunction, oxidative stress, apoptosis, and autophagy. FoxO1, a prominent member of forkhead box O transcription factors, is involved in regulating various cellular processes in different tissues. Altered FoxO1 expression and activity have been associated with cardiovascular diseases in diabetic subjects. Herein we provide an overview of the role of FoxO1 in various molecular mediators related to DbCM, such as altered energy metabolism, lipotoxicity, oxidative stress, and cell death. Furthermore, we provide valuable insights into its therapeutic potential by targeting these perturbations to alleviate cardiomyopathy in settings of type 1 and type 2 diabetes.

## Introduction

Diabetes has evolved exponentially, affects 463 million people worldwide, and prevalence is expected to increase to 700 million by 2045 [1]. Despite numerous advancements in the management of hyperglycemia, cardiovascular diseases including myocardial infarction or heart failure remain the number one cause of death in people with type 1 diabetes (T1D) or type 2 diabetes (T2D) [2]. Although macrovascular dysfunction, endothelial dysfunction, atherosclerosis, and hypertension are increased in diabetic individuals, the increased risk of heart failure is often independent of these comorbidities [3, 4]. Moreover, people with diabetes frequently develop an asymptomatic diastolic dysfunction, a hallmark of diabetic cardiomyopathy (DbCM) [5]. Although the definition of DbCM is still evolving, it is unequivocally considered as ventricular dysfunction with altered myocardial metabolism in the absence of underlying coronary artery diseases and/or hypertension in people with diabetes [5, 6]. Our understanding of pathological changes and molecular mediators of DbCM has greatly improved in last few decades [5], yet there is no approved therapy.

Forkhead box O (FoxO) transcription factors, including FoxO1, FoxO3, FoxO4, and FoxO6, have important roles in several signaling pathways involved in human health and diseases [7]. Out of these subtypes, FoxO1 and FoxO3 are known to be essential for the maintenance of cardiac health by having pivotal roles in the regulations of cellular processes [8]. Lately, with the availability of various pre-clinical models of DbCM, mounting evidence has shown that FoxO1 activity is upregulated in diabetic myocardium [9, 10]. It is also widely accepted that FoxO1 could contribute to the pathogenesis of DbCM via direct or indirect regulations of molecular targets involved in metabolism, oxidative stress, endothelial dysfunction, and apoptosis [10].

In this review, we will provide an overview of the pathology of DbCM and discuss the FoxO1-driven regulations of its key mediators. While our focus will primarily be DbCM in the context of T2D, we will also consider these aspects in the setting of T1D. Furthermore, we will interrogate whether FoxO1 could be a potential therapeutic target for the treatment of DbCM.

## Diabetic cardiomyopathy

The DbCM was first described by Rubler and colleagues in 1972 through findings from an autopsy of four diabetic individuals with no sign of myocardial infarction but with left ventricular (LV) hypertrophy, gross cardiomegaly, and congestive heart failure [11]. These observations led to the very first definition of DbCM, a ventricular dysfunction in the absence of underlying coronary artery disease and/or hypertension in people with diabetes. Although the clinical phenotype of DbCM is still under active investigation, our understanding of it has greatly advanced by utilizing modern non-invasive imaging technology [12, 13]. The growing recognition of diastolic dysfunction and alterations in myocardial metabolism mainly an elevation in fatty acid oxidation and a reduction in glucose oxidation in early-stage T2D is reshaping perspectives of DbCM, re-terming or redefining it as “diabetic heart disease” [5] or “diastolic dysfunction with altered myocardial metabolism without other known causes of cardiomyopathy and/or hypertension” [6]. However, re-terming it as “diabetic heart disease” could mistakenly encompass all cardiovascular conditions linked to diabetes, not just those affecting the myocardium, but also vascular diseases. Moreover, diastolic dysfunction often lacks symptoms and remains undiagnosed in diabetic individuals until a noticeable decline occurs, yet its prevalence in T2D has been reported to range from 20 to 80% based on diagnostic criteria and patient group [14–17]. Indeed, we concur with these perspectives, particularly as diastolic dysfunction and DbCM are significant risk factors for the advancement of heart failure with preserved ejection fraction (HFpEF), which is quite prevalent in individuals with diabetes [18]. Although the advancements in the understanding of DbCM have been greatly appreciated in the last few decades [5, 19], it is still unclear why some individuals with diabetes develop HFpEF whereas others develop heart failure with reduced ejection fraction (HFrEF).

Our understanding of the various mechanisms that contribute to the pathology of DbCM has greatly enhanced with improved knowledge of animal models of obesity and insulin resistance (extensively reviewed by Heather et al. [20]). As of now, we are fully aware of several attenuated cellular processes identified within the myocardium of DbCM subjects. These include lipotoxicity, glucotoxicity, mitochondrial dysfunction, abnormal substrate metabolism, oxidative stress, inflammation, and abnormal calcium handling, many of which can lead to the death of cardiac cells, we encourage the reader to refer to the excellent reviews on this topic [5, 19, 21, 22]. Assessing how these factors individually affect diastolic dysfunction in T2D individuals is challenging due to their cross-talk. For instance, insulin resistance can alter metabolism, leading to mitochondrial dysfunction, oxidative stress, and lipotoxicity [23, 24]. Identifying the most effective target for improving diastolic dysfunction remains uncertain, highlighting the need for future studies to explore these mechanisms in T2D subjects.

## Forkhead box O1 transcription factor (FoxO1)

**FIGURE 1:**
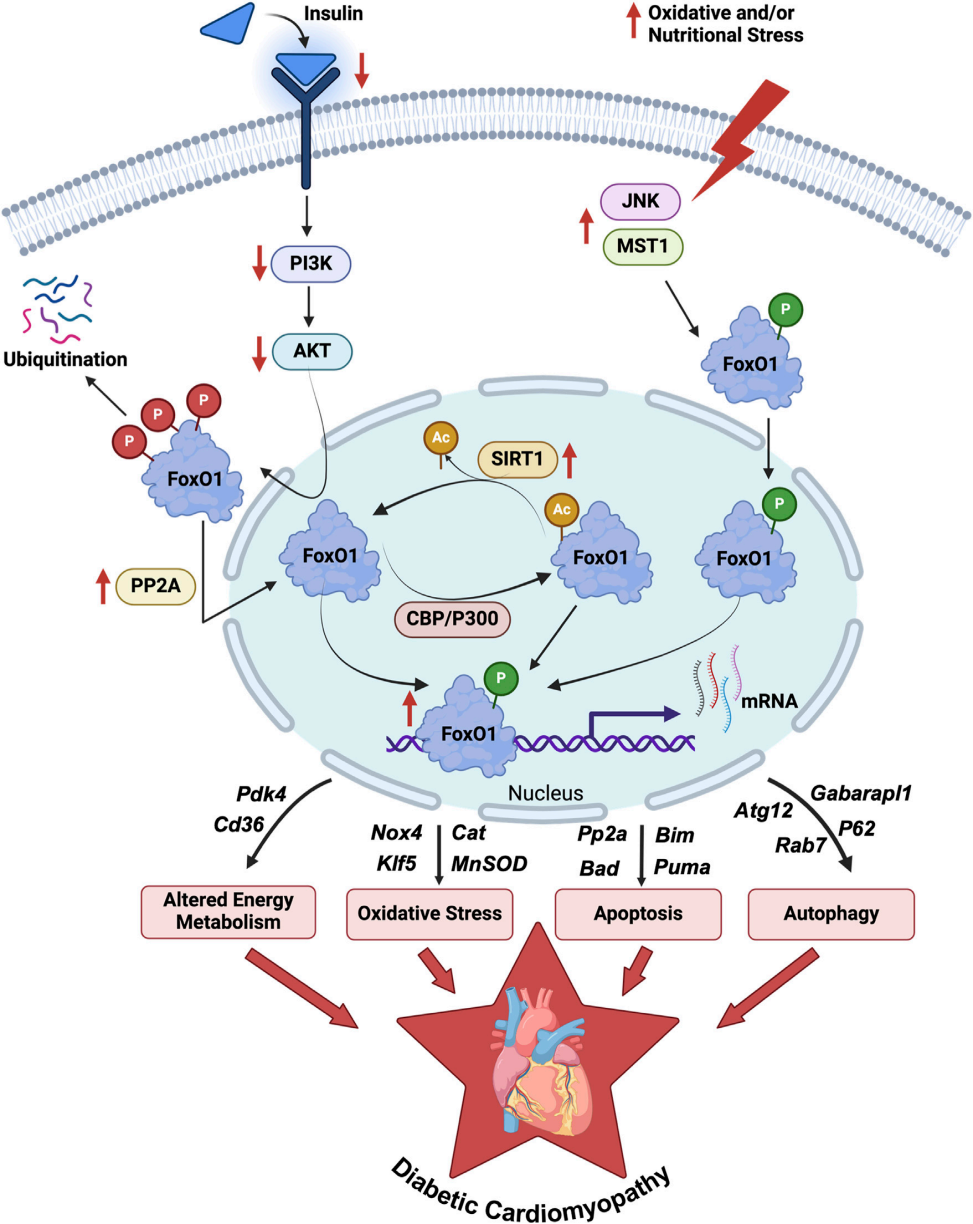
Regulations and role of FoxO1 in DbCM. An illustration depicts the regulation of FoxO1 activity via post-translational modifications such as phosphorylation, acetylation, and ubiquitination in the myocardium of diabetic individuals. The enhanced FoxO1 activity transcriptionally upregulates several genes (Table 1) involved in myocardial energy metabolism, oxidative stress regulations, apoptosis, and autophagy reported in preclinical and clinical studies of DbCM. DbCM, diabetic cardiomyopathy; FoxO1, Forkhead box O1 transcription factor; PI3K, Phosphoinositide 3-kinases; AKT, Protein kinase B; PP2A, Protein phosphatase 2A; CBP, cAMP-response element binding protein; P300, Histone acetyltransferase p300; SIRT1, Sirtuin-1; JNK, c-Jun N-terminal kinase; MST1, Macrophage stimulating 1; Pdk4, Pyruvate dehydrogenase kinase 4; Cd36, fatty acid translocase; Nox4, NADPH oxidase 4; Klf5, Kruppel-like factor 5; Cat, Catalase; MnSOD, Manganese superoxide dismutase; Bim, Bcl2 interacting mediator; Bad, Bcl2 antagonist of cell death; Puma, p53 upregulated modulator of apoptosis; Atg12, Autophagy related 12; Rab7, Rat sarcoma virus-related protein 7; Gabarapl1, Gamma-aminobutyric acid receptor-associated protein-like 1; P62, ubiquitin-binding protein p62; P, Phosphorylation (Red, AKT mediated; Green, JNK1/MST mediated); Ac, Acetylation.

The “Forkhead” protein was first identified in 1989 in *Drosophila melanogaster* as a transcriptional regulator containing a winged-helix DNA binding domain [25, 26]. Later in the 1990s, FoxO was identified as abnormal dauer formation-16 (DAF-16) in *Caenorhabditis elegans* and as forkhead in rhabdomyosarcoma (FKHR) in tumor tissues from eight patients with alveolar rhabdomyosarcomas [27, 28]. In humans, there are four FoxO proteins including FoxO1, FoxO3, FoxO4, and FoxO6 are known to be present in various tissues [29, 30]. Although FoxO6 was initially thought to be mainly in the brain, it is now known for a ubiquitous expression as well [31]. FoxO1/3/4/6 proteins through their conserved forkhead domain specifically recognize DAF-16 binding element (DBE) 5′-GTAAACAA-3′ and insulin-responsive element (IRE) 5´-(C/A)(A/C)AAA (C/T)AA-3′ to transcriptionally regulate the expression of genes (extensively reviewed in [32, 33]). FoxO’s nuclear transit and transcriptional activity are also regulated by various post-translational modifications such as phosphorylation, acetylation, O-glycosylation, methylation, and ubiquitination. Although several kinases (e.g., mitogen-activated protein kinases, c-Jun N-terminal kinases, cyclin-dependent kinase 2, nuclear factor κB, etc.) are known to be involved in the phosphorylation of FoxO1, protein kinase B (also known as AKT), a downstream target of insulin signaling have been considered a prime kinase which negatively regulates FoxO1 by phosphorylation and nuclear exclusion in the context of metabolism (Figure 1) [34, 35]. In the 21st century, a plethora of studies concluded the essential role of FoxO transcription factors in myocardial homeostasis through the regulation of cell proliferation, oxidative stress, energy metabolism, and cell death (extensively reviewed in [36, 37]). FoxO1, among other “O” subfamilies, has been considered the front-runner in controlling myocardial equilibrium in the settings of metabolic diseases (especially in DbCM) [10, 36, 38].

## FoxO1 in diabetic cardiomyopathy

The plethora of evidence suggests that the pathophysiology of DbCM [9, 39, 40], and ischemic heart disease [41] are linked to upregulated FoxO1 activity. Although there is no direct clinical evidence of FoxO1 activation during DbCM, its DNA binding sites are overrepresented in the promoter sequences of heart failure genes in isolated RNA from the myocardium of heart failure patients with either ischemic or idiopathic dilated cardiomyopathy [42]. Additionally, RNA sequencing data of human hearts with dilated cardiomyopathy showed an enriched FoxO1-binding motif, suggestive of enhanced transcriptional activity [43]. However, the contribution of cellular mechanisms associated with FoxO1 signaling in the pathogenesis of DbCM is not yet fully understood. The connection between FoxO1 activation and the pathogenesis of DbCM mainly stemmed from *in vivo* animal models and *in vitro* studies [9, 44]. In the case of insulin resistance and diabetes, reduced growth signals and increased stress signals lead to weaker nuclear exportation mechanisms for FoxO1, resulting in increased FoxO1 transcriptional activity in cardiomyocytes [36]. The increased transcriptional activity of FoxO1 precipitates shifts in gene expression, consequently inducing modifications in myocardial energy metabolism, lipotoxicity, oxidative stress, and cellular damage in diabetic myocardium (Figure 1; Table 1).

**TABLE 1:** Potential effects of FoxO1 regulation in DbCM.

Animal models	Intervention/Targets	Effects	References
HFD-fed obese mice	Cardiac-specific FoxO1 knockout	↓ Myocardial TAG content ↓ LV Hypertrophy ↓ Cardiac systolic dysfunction Improves myocardial PDH activity	[9]
HFD and STZ-induced T2D mice	FoxO1-*Pdk4* axis targeted with AS1842856 and cardiac-specific FoxO1 knockout	↑ Myocardial glucose oxidation ↓ Diastolic dysfunction	[45, 46]
STZ-induced T1D Sprague-Dawley rats	FoxO1 inhibition by AS1842856	Improves cardiac function ↑ Glucose oxidation ↓ Apoptosis	[47]
Wistar rats with lipid overload	FoxO1-iNOS-CD36 pathway	Cardiomyocyte lipid accumulation	[48]
Db/db mice	FoxO1-CD36 axis targeted with Evogliptin	Protects against DbCM Prevents lipotoxicity	[49]
HFD-Fed mice	FoxO1-CD36 axis EP4-deficient mice	↓ Myocardial fatty acid uptake ↓ ATP production	[50]
STZ-induced T1D mice	FoxO1-KLF5 Cardiac-specific FoxO1 knockout	↓ Oxidative stress ↓ Cardiac dysfunction	[51]
Sprague-Dawley rats on high-glucose and HFD with STZ	Sirt1-FoxO1 and PI3K-Akt signaling pathways targeted with Curcumin	↓ Oxidative stress ↓ Apoptosis ↓ DbCM	[52]
Db/db mice	Akt-FoxO1 signaling by Diazoxide	↓ Apoptosis	[53]
STZ-induced T1D mice	Angiotensin IV AS1842856	↓ Autophagy	[54]
STZ-induced T1D mice	Resveratrol FoxO1- Rab7	Restores Autophagic flux	[55]

### FoxO1 in metabolic abnormalities during diabetic cardiomyopathy

Numerous studies have consistently affirmed the idea that disruptions in myocardial glucose and fatty acid metabolism serve as primary triggers for cardiac dysfunction in diabetic conditions, a topic thoroughly reviewed by Heather et al. [6]. FoxO1 is involved in various pathways related to myocardial energy metabolism. Battiprolu et al. have shown that 25 weeks of high-fat diet (HFD) (60% kcal from lard) feeding to male C57BL/6J mice induces myocardial nuclear enrichment of FoxO1, leading to enhancement in myocardial triacylglycerol (TAG) content, LV hypertrophy, and cardiac systolic dysfunction, which was not apparent in HFD-fed cardiac-specific FoxO1 deficient mice [9]. In addition, enhanced FoxO1 nuclear compartmentalization contributed to elevations in myocardial pyruvate dehydrogenase (PDH) kinase 4 (*Pdk4*) transcription and impairment in PDH activity in the myocardial tissues of HFD-fed mice. Concurrently, we have shown that FoxO1 binds to the DBE sequence in the promoter of the *Pdk4* gene to upregulate its expression in cardiomyocytes and reduce myocardial glucose oxidation rates [45]. As the glucose oxidation produces more ATP per mole of consumed oxygen than the fatty acid oxidation, reduced cardiac function correlated with higher oxygen consumption and lower cardiac efficiency in *ob/ob* mice with reduced glucose oxidation and increased fatty acid oxidation [56, 57]. Additionally, the increases in the myocardial delivery of fatty acids due to adaptive changes may lead to the uncoupling of the mitochondria, leading to a reduction in ATP production which aligns with the reduced cardiac performance. Moreover, recent studies from our lab targeting this FoxO1-*Pdk4* axis using either AS1842856 (FoxO1 inhibitor) or cardiac-specific FoxO1 elimination alleviated diastolic dysfunction via increasing myocardial glucose oxidation rates in male mice subjected to experimental T2D via HFD supplementation for 10 weeks with a single dose (75 mg/kg) of streptozotocin (STZ) at 4th week [45, 46]. Similarly, male Sprague Dawley rats induced with T1D using STZ (65 mg/kg) and treated with AS1842856 demonstrated improved cardiac function using pressure-volume conductance catheters [47]. The isolated cardiomyocytes from these rats demonstrated increased oxygen consumption rates in the presence of glucose or pyruvate (indicative of increased glucose oxidation). These findings strongly advocate the role of FoxO1 in the reduction of glucose oxidation in the myocardium of diabetic mice with cardiac dysfunction.

In diabetic myocardium, decreases in glucose oxidation with elevated PDK4 expression often result in increases in fatty acid uptake and oxidation by following Randle’s cycle to meet constant energy demand, thereby promoting myocardial lipid accumulation [22]. Contrarily, mice with cardiac-specific overexpression of PDK4 were protected against HFD-induced myocardial lipid accumulation, likely due to adaptive metabolic re-programming for increased fatty acid oxidation [58]. However, Elevated myocardial TAG content-associated lipotoxicity has been verified in individuals with T2D and identified as an independent predictor of diastolic dysfunction [59]. A key early development in DbCM pathogenesis involves increased fatty acid transport across the sarcolemma, primarily controlled by fatty acid translocase (FAT/CD36) [60]. In conditions of lipid overload, the FoxO1/inducible NO-synthase (iNOS)/CD36 pathway was shown to mediate lipid accumulation in cardiomyocytes from adult male Wistar rats [48]. Palmitate exposure in isolated cardiomyocytes leads to a significant overload of intercellular TAG which triggers a chain reaction starting with the upregulation of FoxO1. The high expression of FoxO1 in the vascular endothelial cells leads to an overexpression of iNOS which activates the cell division control (Cdc) 42 protein through its nitration, resulting in cytoskeleton rearrangement. This process aids CD36 translocation and results in TAG accumulation in cardiomyocytes from adult male Wistar rats [48]. Moreover, Evogliptin (EVO), a dipeptidyl peptidase-4 (DPP-4) inhibitor known for its glucose-lowering effects in T2D, demonstrated the ability to prevent DbCM and associated lipotoxicity by suppressing CD36 protein expression and enhancing the phosphorylation of FoxO1 at Serine 256 position, indicative of its inactivation, in db/db mice [49]. Prostaglandin E receptor subtype 4 (EP4) is a G protein-coupled receptor (GPCR) highly expressed in cardiomyocytes. In a study involving mice supplemented with HFD for 8 weeks, EP4 was shown to protect against DbCM by modulating FoxO1/CD36-mediated fatty acid uptake [50]. The concentric hypertrophy and myocardial fibrosis in HFD-fed EP4-deficient mice converged with a reduction in myocardial fatty acid uptake and ATP production, which was corrected pharmacologically by activation of EP4. Thus, by targeting the FoxO1–CD36 axis, we could reduce the myocardial damage associated with lipotoxicity during diabetes.

### FoxO1 in myocardial oxidative stress during diabetic cardiomyopathy

It is undebatable that hyperglycemia along with enhanced fatty acid oxidation and mitochondrial dysfunction contributes to oxidative stress by increasing reactive oxygen species (ROS) including superoxide and H_2_O_2_ levels in the diabetic myocardium [61]. FoxO1 has been known to play a dual role during oxidative stress regulation based on the cellular microenvironment and level of oxidative stress [37]. Recently, Krüppel-like factor (KLF) 5 directly transcriptionally regulated by FoxO1 was shown to cause oxidative stress via induction of NADPH oxidase (NOX) 4 expression, a major source of cytosolic ROS levels [62] in cardiomyocytes of STZ-induced T1D mice [51]. Cardiac-specific FoxO1 elimination remarkably reduced KLF5 expression and prevented oxidative stress and cardiac dysfunction, which was reverted by over-expression of FoxO1 or KLF5 in cardiomyocytes of T1D mice. Concurrently, Curcumin, a natural antioxidant, treatment in male Sprague-Dawley rats fed a high-glucose and HFD (40% fat, 41% carbohydrates, and 18% protein) and supplemented with STZ (60 mg/kg; 3 days) was shown to alleviate oxidative stress and DbCM by FoxO1 modulation via sirtuin 1 (Sirt1) and phosphoinositide 3-kinases (PI3K)-AKT signaling pathways [52]. Moreover, high glucose upregulated thioredoxin (Trx) interacting protein (Txnip) expression by binding of FoxO1 to its promoter and subsequently inhibited Trx activity in human aortic endothelial cells [63]. These effects were Trx system-mediated reduction of oxidized cysteine groups on proteins through an interaction with the redox-active center of Trx and activated FoxO1 pathway.

On the other hand, FoxO1 may also protect against oxidative stress in cardiomyocytes by promoting the expression of antioxidant enzymes such as catalase (CAT) and manganese superoxide dismutase (MnSOD) via Yes-associated protein (YAP) pathways to neutralize ROS [64]. STZ‐induced diabetic rats with myocardial metabolism and functional abnormalities showed oxidative stress by reduced activity of SOD, and elevated malondialdehyde [MDA] levels [52]. Curcumin treatment in these rats rescued the activity of SOD by restoring Sirt1-FoxO1 signaling, resulting in reduced ROS and alleviation of DbCM. Moreover, Exenatide, a glucagon-like peptide-1 (GLP-1) receptor agonist, attenuated ROS production through increases in expression of MnSOD and catalase in cardiomyocytes of HFD-fed T2D mice and STZ-induced T1D mice [65]. These protective actions might be mediated through Sirt1-FoxO1 pathways, as the cardioprotective effects of Exendin-4 against ischemia/reperfusion (I/R) injury in male rats involves upregulated activity Sirt1-FoxO1 pathways and associated MnSOD production [66]. Thus, in varying microenvironments such as the level of stress in various cell types of diabetic myocardium or ROS-mediated signaling activation, FoxO1 may play a destructive rather than protective role during oxidative stress regulations [67]. In diabetic myocardium, conditions like hyperglycemia, insulin resistance, and metabolic disturbances such as elevated serum glucose or lipids can induce FoxO1 expression, shifting its function from antioxidant to prooxidant.

### FoxO1 in diabetic cardiomyopathy-associated myocardial cell death

Apart from its roles in energy metabolism and oxidative stress, FoxO1 also has substantial roles in myocardial cell death via apoptosis and autophagy during diabetes [68]. In diabetic myocardium, upregulated FoxO1 activity stimulates the expression of various proapoptotic regulators such as B-cell lymphoma 2 (Bcl2)-associated agonist of cell death (BAD), Bcl-2 Interacting Mediator (BIM), Puma, and caspases [46, 48]. Puthanveetil et al. demonstrated that FoxO1 regulates BAD via up-regulation of protein phosphatase 2A (PP2A) in the diabetic myocardium [48]. However, in cardiomyocytes, overexpression of the wild-type or constitutively active form of FoxO1 has been associated with inhibition of the PP2A/B activity and attenuation of insulin signaling [69]. This divergence likely stems from FoxO1/CD36-mediated lipid buildup in diabetic cardiomyocytes, which may reactivate PP2A and trigger BAD activation, similar to how ceramides stimulate PP2A during arterial dysfunction in obese mice [70]. It is noteworthy that, myocardial apoptosis may not be regulated by FoxO1 in all available pre-clinical models of DbCM. Our recent study demonstrated the attenuation of diastolic dysfunction and altered myocardial metabolism but no effect on apoptosis by pharmacological or genetic inhibition of FoxO1 in T2D mice [46]. However, FoxO1 inhibition by AS1842856 in T1D male Sprague Dawley rats mitigated the apoptosis, evident by a reduction in cleaved caspase 3 expression and tunnel staining [47]. Similarly, curcumin was shown to alleviate apoptosis in cardiomyocytes which was associated with inhibition of FoxO1 acetylation and modulation of Sirt1-FoxO1 signaling in STZ-induced T2D rats [52]. Moreover, the opening of mitochondrial ATP-sensitive potassium (mitoKATP) channels by diazoxide was found to improve cardiac function and attenuate cardiomyocyte apoptosis in db/db mice [53]. The protective effect of diazoxide was associated with a reduction in AKT-FoxO1 signaling and the activity of caspase 3 in cardiomyocytes.

While the significance of autophagy in DbCM is still subject to debate, FoxO1 has been implicated in its regulation. In starvation, FoxO1 can activate the expression of autophagic genes such as autophagy-related protein 12 (Atg12) and γ-aminobutyric acid receptor-associated protein-like 1 (Gabarapl1) in cardiomyocytes [71]. Similarly, glucose-deprived cultured cardiomyocytes showed increased autophagic flux accompanied by Sirt1-associated FoxO1 deacetylation and a decreased expression of ubiquitin-binding protein p62 [72]. Contrarily, acetylated FoxO1 has been shown to upregulate autophagy in a transcription-independent manner by interacting with Atg7 in the cytosol of cancer cells [73]. FoxO1 also plays an essential role in the regression of cardiac hypertrophy via upregulating autophagy during mechanical unloading by reversal of transverse aortic constriction (TAC) in mice [74]. Similarly, FoxO1 contributes to exercise-induced physiological hypertrophy by regulating autophagy markers independent of the PI3K-AKT signaling [75]. Moreover, cardiac-specific overexpression of FoxO1 in transgenic mice exhibited a decrease in the size of hearts and upregulation of autophagy. Concurrently, it has been demonstrated that the cardioprotective effect of angiotensin (Ang) IV in T1D mice was through suppression of FoxO1-induced excessive autophagy [54]. The protective effects of Ang IV were completely blocked by over-expression of FoxO1, which was reversed by the additional administration of AS1842856. However, resveratrol has been shown to protect against DbCM by restoring autophagic flux [55]. The effect was achieved through the upregulation of FoxO1-mediated transcription of rat sarcoma virus-related protein (Rab)7, a small GTP-binding protein that mediates late autophagosome-lysosome fusion. Thus, the enhancement of FoxO1 activity contributes to dysregulated apoptosis and autophagy in diabetes, and targeting these perturbations could alleviate the progression of DbCM.

## Discussion

Taken together, FoxO1 dysregulations could exacerbate damages in myocardial cellular processes, accelerating the development of diastolic dysfunction during DbCM, a major complication in people with diabetes. Metabolic alterations, oxidative stress, and cell death are implicated in both the progression of DbCM and the regulatory processes involving FoxO1. Enhanced FoxO1 expression and activity appear to promote alteration in myocardial glucose and fatty acid metabolism, oxidative stress, and cell death in DbCM. Notably, the above-discussed findings are mainly based on animal models of T1D or T2D and clinical applications of FoxO1 signaling in cardiac injury in DbCM are still unknown. Moreover, the interplay between different molecular mediators of DbCM and their regulation by FoxO1 in pre-clinical models is largely unknown to predict a translational aspect of these findings. Thus, we currently lack enough information on whether FoxO1 or its pathways could be a therapeutic target during DbCM in people with diabetes. A promising approach could be the optimization of cardiac energy metabolism, though an improved understanding of how FoxO1-mediated modulations of myocardial energy metabolism, oxidative stress, cell death, and its interplay regulate diastole may direct us to better molecular targets for future drug development.
